# Human DDX3X Unwinds Japanese Encephalitis and Zika Viral 5′ Terminal Regions

**DOI:** 10.3390/ijms22010413

**Published:** 2021-01-02

**Authors:** Corey Nelson, Tyler Mrozowich, Darren L. Gemmill, Sean M. Park, Trushar R. Patel

**Affiliations:** 1 Department of Chemistry and Biochemistry, Alberta RNA Research and Training Institute, University of Lethbridge, 4401 University Drive, Lethbridge, AB T1K 3M4, Canada; corey.nelson@uleth.ca (C.N.); tyler.mrozowich@uleth.ca (T.M.); d.gemmill@uleth.ca (D.L.G.); sean.park@uleth.ca (S.M.P.); 2 Department of Microbiology, Immunology and Infectious Disease, Cumming School of Medicine, University of Calgary, Calgary, AB T2N 1N4, Canada; 3 Li Ka Shing Institute of Virology and Discovery Lab, University of Alberta, Edmonton, AB T6G 2E1, Canada

**Keywords:** DDX3X, Japanese encephalitis virus, Zika virus, viral terminal regions, host–viral interactions, in vitro transcription, microscale thermophoresis, RNA helicase assays

## Abstract

Flavivirus genus includes many deadly viruses such as the Japanese encephalitis virus (JEV) and Zika virus (ZIKV). The 5′ terminal regions (TR) of flaviviruses interact with human proteins and such interactions are critical for viral replication. One of the human proteins identified to interact with the 5′ TR of JEV is the DEAD-box helicase, DDX3X. In this study, we in vitro transcribed the 5′ TR of JEV and demonstrated its direct interaction with recombinant DDX3X (K_d_ of 1.66 ± 0.21 µM) using microscale thermophoresis (MST). Due to the proposed structural similarities of 5′ and 3′ TRs of flaviviruses, we investigated if the ZIKV 5′ TR could also interact with human DDX3X. Our MST studies suggested that DDX3X recognizes ZIKV 5′ TR with a K_d_ of 7.05 ± 0.75 µM. Next, we performed helicase assays that suggested that the binding of DDX3X leads to the unwinding of JEV and ZIKV 5′ TRs. Overall, our data indicate, for the first time, that DDX3X can directly bind and unwind in vitro transcribed flaviviral TRs. In summary, our work indicates that DDX3X could be further explored as a therapeutic target to inhibit Flaviviral replication

## 1. Introduction

Infection with pathogenic viruses often leads to severe diseases that may impact, among others, the metabolic, respiratory, digestive, and central nervous systems. Among the most pathogenic family of viruses, emerging and re-emerging outbreaks of flavivirus are responsible for thousands of deaths annually [1]. Furthermore, flaviviral infections also lead to significant morbidities in survivors, which can create a substantial burden on the health system [2,3]. The Flaviviridae family includes the most prevalent arthropod-borne viruses such as dengue, Japanese encephalitis (JEV), Murrey Valley, Powassan, West Nile (WNV), yellow fever, and Zika (ZIKV) viruses. Flaviviral outbreaks are becoming increasingly common, due to the ease of transmission by mosquitoes and the lack of efficient therapeutics or immunoprophylactic strategies [4,5,6]. As a result, flaviviruses are emerging as a global health threat. For example, the WHO reports that since 2015, ZIKV outbreaks have been reported in 42 countries [7]. The majority of flaviviruses utilize *Aedes* and *Culex* genuses of mosquitoes for transmission [8], meaning that as global temperatures increase, countries that were once protected from arboviruses are also becoming increasingly at risk [9,10]. 

The JEV is responsible for approximately 68,000 cases annually, with a fatality rate between 20–30% and with 30% of cases developing serious long-term disabilities [11,12]. This makes JEV one of the deadliest flaviviruses, although an approved vaccine against JEV is available [13,14]. The Zika virus (ZIKV) outbreak infected >4.5 million people in Brazil and the Americas in 2015–2016 [15,16]. ZIKV is also linked to birth defects [3,17,18,19,20,21,22,23] and neurological disorders [24,25,26,27,28,29,30]. The ZIKV has also been observed to be transmitted sexually and present symptoms in only one-fifth of cases [31,32]. Unlike JEV, there is no approved vaccine available against ZIKV. Attempts to develop vaccines have faced unexpected challenges due to antibody-dependent enhancement of infection with other flaviviruses [33,34,35]. Flaviviruses contain a positive-sense single-stranded RNA genome, comprised of 5′ and 3′ untranslated terminal regions (TRs). The 5′ TRs (~0.1 kb) contain a type 1 capped structure, whereas the 3′ TRs (~0.3 to ~0.5 kb) lack a poly(A) tail, and both TRs include conserved structural motifs [36,37,38,39,40,41]. The interactions between flaviviral 5′ and 3′ TRs are also critical for viral replication [6,41,42,43,44,45,46]. These regions flank a single open reading frame (ORF), which encodes a single polypeptide that is cleaved by a combination of host and viral proteases [47]. Previous work has established that interactions of host proteins with the flaviviral TRs are crucial for viral replication [48,49,50,51,52,53,54,55,56,57,58,59]. 

**Figure 1:**
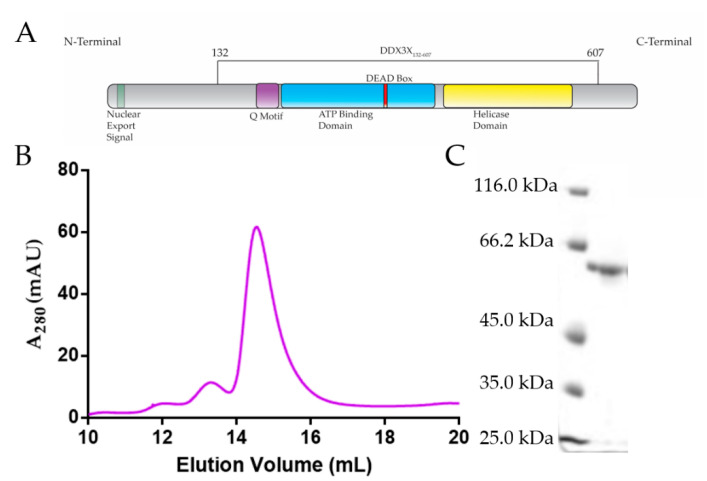
Purification of recombinant DDX3X_132–607_. (**A**) Schematic of human DDX3X’s domain architecture, indicating that DDX3X_132–607_ consists of all the major domains, except the nuclear export signal sequence. (**B**) Size exclusion chromatography purification (Superdex 200 Increase GL 10/300) of DDX3X_132–607_ demonstrating that DDX3X_132–607_ can be purified to homogeneity, eluting at ~14.5 mL. Y-axis represents absorbance at 280 nm while the x-axis represents elution volume. (**C**) SDS-PAGE indicating that the size exclusion chromatography (SEC)-purified DDX3X_132–607_ is monodispersed with the correct molecular weight (55.3 kDa).

The human DEAD-box family of helicases is comprised of 37 members, each composed of conserved helicase core domains that interact with ATP and RNA [60,61]. The DEAD-box helicases consist of two helicase domains; domain 1 containing motifs Q, I, Ia Ib, Ic, II (DEAD-box), and III; and domain 2 containing motifs IV, IVa, V, Va, and VI [61]. These motifs are involved in either ATP binding/hydrolysis, RNA binding, or couple ATP and RNA binding activities. Although the classical function of DEAD-box helicases is ATP-dependent unwinding of nucleic acids [62], they influence all major aspects of RNA metabolism [63]. DDX3X (X-linked DDX3, 73 kDa, Figure 1A) is one of the ATP-dependent RNA helicases that plays critical roles in transcription, translation, and mRNA (messenger-RNA) export [59,64,65]. DDX3X unwinds RNA in an ATP-dependent manner, where DDX3X binds to dsRNA (double-stranded RNA) and hydrolyzes ATP to release single-stranded RNA [66]. Apart from playing vital roles in cellular activities, DDX3X was shown to suppress dengue viral infection via interferon activation [67] but promotes WNV infection [68]. Using affinity pull-down and Western blot analysis, it was suggested that DDX3X could interact with 5′ TR of JEV and regulate its replication [50]. Recent studies have suggested that DDX3X inhibitors can suppress WNV replication [68]. Given the magnitude and severity of flaviviral infections, there is a critical need for therapeutics; however, their development is hindered by the limited understanding of the interactions of viral RNAs with the host cellular proteins.

In this study, we followed up Li et al.’s affinity pull-down assays and demonstrated that DDX3X directly interacts with the 5′ TR of JEV [50]. As the 5′ TRs of flaviviruses are hypothesized to be structurally similar [69,70], we asked if the 5′ TR of ZIKV can also be recognized by DDX3X. Our binding studies demonstrated that DDX3X indeed interacts with the 5′ TR of ZIKV. As DDX3X is an RNA helicase, we performed helicase assays, which suggested that both JEV and ZIKV 5′ TR can be unwound by DDX3X. In summary, our study highlights that DDX3X could serve as an important therapeutic target to inhibit JEV and ZIKV replications. 

## 2. Results

### 2.1. Purification of DDX3X_132–607_, JEV 5′ and Zika 5′ TR RNAs

The DDX3X_132–607_ was expressed in Lemo21(DE3) Esherichia coli cells, followed by initial purification using affinity chromatography. Subsequently, affinity-purified DDX3X_132–607_ was subjected to size exclusion chromatography (SEC) purification (see section 4 for additional details). As presented in Figure 1B, we were able to remove minor aggregation at ~12 mL and ~13 mL elution volumes to obtain homogenous preparation of DDX3X_132–607_ (peak at ~14.5 to 15.5 mL). Subsequently, we analysed the peak fractions using SDS-PAGE, which suggested that DDX3X_132–607_ is devoid of any degradation (Figure 1C, right lane) and the amino-acid sequence-based molecular weight of 55.3 kDa for DDX3X_132–607_ coincides with the observed band in Figure 1C. 

**Figure 2:**
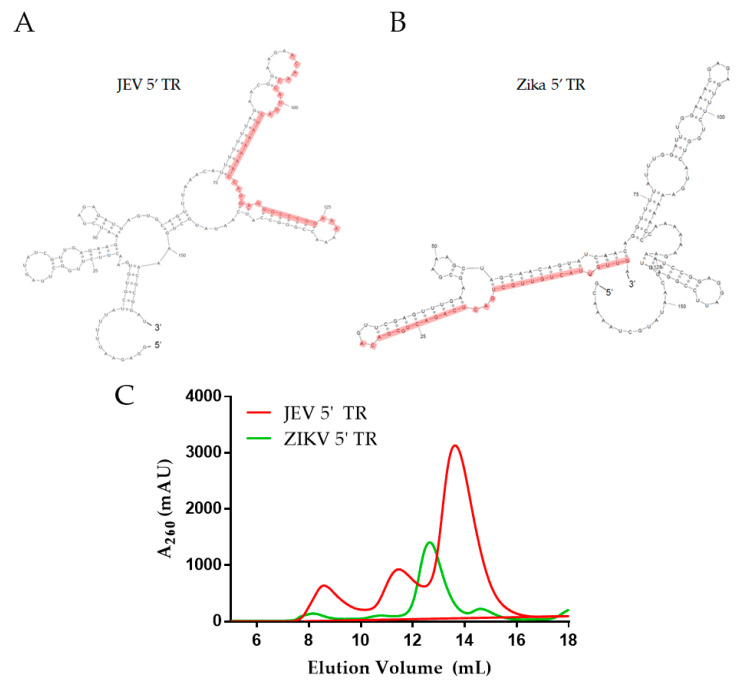
*(***A** and **B**) Predicted secondary structure (sfold) for the 5′ terminal regions (TRs) of Japanese encephalitis virus (JEV) 5′ and Zika virus (ZIKV), respectively. The regions highlighted in red colour indicate the sequence complementary to the DNA oligo used in the helicase assays. (**C**) Size exclusion chromatography elution profiles of the 5′ TRs of JEV (red) and ZIKV (green). Arrows indicate peaks that represent monodispersed fractions of RNA, which were used for downstream experiments. Y-axis represents absorbance at 260 nm while the x-axis represents elution volume.

The predicted secondary structure of 5′ TRs of JEV and ZIKA (Figure 2A,B) indicated that both RNAs are composed of a significant amount of double-stranded regions, along with stem-loops. The 5′ TRs for both viruses were in vitro transcribed and natively purified using SEC, similarly to DDX3X_132–607_. The SEC purification indicated that while the ZIKV 5′ TR RNA elutes at approximately 12.5 mL, the JEV 5′ TR elutes at ~13.8 mL (Figure 2C). In both cases, oligomeric or aggregated species appear to elute around ~10.0 to 11.5 mL. In both SEC profiles, the plasmid DNA that was used as a template elutes around 8 mL, consistent with the column’s void volume. Urea-PAGEs confirmed that both RNAs were purified to homogeneity (Supplementary Figure S1). Monodispersed peak fractions(s) were used in downstream experiments.

### 2.2. DDX3X_132–607_ Binds to 5′ TRs of JEV and ZIKV

**Figure 3:**
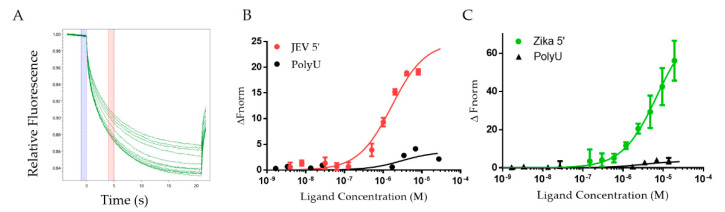
Interaction studies using microscale thermophoresis (MST). (**A**) Representative MST traces depicting the change in fluorescent migration of fluorescein-5-thiosemicarbazide (FITC)-JEV 5′ TR due to the excitation with an infra-red laser. Each green trace correlates to a different concentration of DDX3X_132–607_. (**B**) Binding data for DDX3X_132–607_ with JEV 5′ TR (*n* = 3). The red curve represents JEV 5′ and has a dissociation constant of 1.66 ± 0.21 µM (Std. error of regression = 1.25). The black trace represents that polyU (negative control) does not interact with DDX3X_132–607_. (**C**) Interaction between DDX3X_132–607_ and ZIKV 5′ TR (*n* = 3) studied using MST. The green curve represents ZIKV 5′ and has a dissociation constant of 7.05 ± 0.75 µM (Std. error of regression = 1.22). PolyU binding data included for reference (black).

To determine the binding affinity of a previously uncharacterized interaction system containing DDX3X_132–607_ and 5′ TR of JEV, we employed microscale thermophoresis (MST) as performed previously [71,72]. DDX3X_132–607_ was titrated against the constant concentration of the fluorescent ncRNAs (non-coding RNAs) to determine their dissociation constant (K_d_). Figure 3A displays the change in fluorescent migration when the infrared laser affects the samples, where each trace represents a different concentration of DDX3X_132–607_. Additionally, the blue bar indicates the “cold” region, and the red bar indicates the “hot” region. Figure 3B,C present the binding curves for 5′ TRs of JEV and ZIKV in red and green, respectively. These experiments suggest that DDX3X_132–607_ has a K_d_ of 1.66 ± 0.21 µM for the 5′ TR of JEV and 7.05 ± 0.75 µM for the 5′ TR of ZIKV. 

### 2.3. DDX3X_132–607_ Unwinds 5′ TRs of JEV and ZIKV

**Figure 4:**
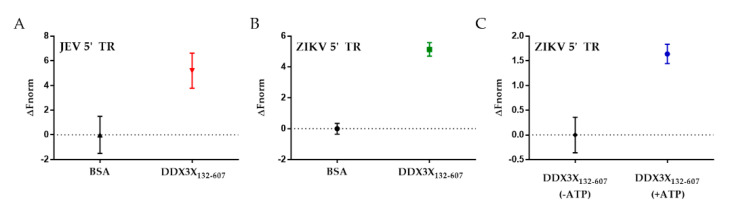
Helicase assays conducted using microscale thermophoresis (MST). (**A**) Comparing the change of fluorescent migration of a complementary DNA oligo in the presence of 5′ TR of JEV with ATP and either bovine serum albumin (BSA) or DDX3X_132–607_. The average signal to noise ratio is 14.8 ± 4.82 (*n* = 3). (**B**) Comparing the fluorescent migration of a complementary DNA oligo in the presence of ZIKV 5′ TR with ATP and either BSA or DDX3X_132–607_. The average signal to noise is 20.0 ± 5.06 (*n* = 3). (**C**) Investigating the role of ATP in DDX3X_132–607_ helicase activity. The average signal to noise ratio is 5.5 ± 0.59 (*n* = 3), suggesting that ATP is required to unwind RNA.

To further investigate if the binding of flaviviral 5′ TRs with DDX3X_132–607_ leads to their unwinding, we designed a helicase assay using MST. We hypothesized that if DDX3X_132–607_ unwinds the 5′ TRs of JEV and ZIKV, the newly formed single-stranded region of viral RNAs could hybridize with their complementary DNA oligos. Therefore, we designed fluorescently-labelled DNA oligos complementary to the region highlighted in red colour in Figure 2A,B for JEV and ZIKV, respectively. We incubated 5′ TR of JEV (or ZIKV) with DDX3X_132–607_ (or bovine serum albumin (BSA) as a negative control), ATP and complementary oligos, followed by measurements of MST traces. Our MST experiments suggest that in the presence of DDX3X_132–607_, the oligos migrated differently than in the presence of BSA. This indicates that DDX3X_132–607_ is able to unwind the double-stranded region, allowing the oligo to bind. Figure 4A,B present the unwinding of 5′ TRs of JEV and ZIKV with a signal to noise ratio of 14.8 ± 4.82 and 20.0 ± 5.06, respectively. Note that the signal to noise ratio of 12 or higher indicates the excellent quality of the assay [73]. To achieve the effect of a null mutant DDX3X, which can involve mutating the “DEAD” region in the ATP-binding domain [74], we compared the ability of DDX3X_132–607_ to unwind ncRNA in the presence and absence of ATP. Figure 4C shows the change in fluorescent migration as a result of ATP. Adding ATP caused a significant change in the migration of the DNA oligos, indicating that the unwinding activity increased in the presence of ATP.

## 3. Discussion

The purification of DDX3X_132–607_ (55.3 kDa) through SEC resulted in the protein eluting at ~14.5 mL (Figure 1B), which is consistent with our previous purification of a similar molecular weight human helicase, DDX17_135–555_ (48.5 kDa) [75]. As both helicases are highly similar and belong to the same family of proteins, we are confident that the peak we observe for DDX3X_132–607_ is consistent with a homogenous, monodispersed preparation. Further quality control was performed through SDS-PAGE, which confirms that the peak SEC fractions contain a singular monodispersed species at the correct molecular weight, and are devoid of any degradation (Figure 1C). The sfold predicted secondary structures reveal that each RNA adopts a high degree of double-stranded regions (Figure 2A,B), which is consistent with previous reports of highly structured flaviviral terminal regions [70]. 

RNA purification was performed immediately after in vitro transcription reaction using SEC. The 5′ TRs of JEV (159 nts) and Zika (163 nts) have sequence molecular weights of 51.5 and 52.3 kDa, respectively. However, they elute at distinctly different positions during SEC purification (Figure 1C). We believe that the differences in their structures lead to their elution at different positions. For example, based on its predicted structure, it appears that the 5′ TR of JEV could adopt a more compact structure compared to the 5′ TR of ZIKV (Figure 2A,B). The relatively extended conformation of 5′ TR of ZIKV will have a larger hydrodynamic radius compared to that of JEV, which will cause it to elute earlier in SEC. These results lead us to believe that both RNAs are folded correctly, whereas if the RNA were denatured, we would expect the elution peak to be virtually identical for both RNAs. The MST studies demonstrate that the JEV 5′ TR interacts with high affinity with DDX3X, compared to the ZIKV 5′ TR. As both ncRNAs are of same size but have different conformations, as suggested by SEC elution profiles (Figure 2C), it could be speculated that DDX3X has high affinity for compact conformation, compared to an extended one. However, the precise mechanisms that determine the specificity and the biological relevance of DDX3X-ZIKV interactions require additional work. 

DEAD-box helicase DDX3X has been implicated in many viral systems as a key regulator of viral replication [50,67,68]. However, we lack insights into the affinity of DDX3X for any viral RNA. Moreover, whether DDX3X unwinds viral RNA is also unclear. Therefore, we utilized MST that has emerged as one of the ideal techniques to study biomolecular interactions [76,77]. By assessing the change in fluorescent migration under the influence of an infra-red laser, we are able to quantify the binding affinity between DDX17_135–555_ and 5′ TRs. To our knowledge, this work provides the first evidence of DDX3X directly interacting with in vitro transcribed, natively purified viral terminal regions, as well as the binding affinity of DDX3X with viral RNAs. Figure 3B,C also highlight how two different viral RNA TRs can have different affinities, despite being highly conserved sequences [70]. The 5′ TR of JEV has over four-times stronger affinity for DDX3X_132–607_ compared to the 5′ TR of ZIKV. Furthermore, DDX3X was identified as an interacting partner for the 5′ TR of JEV in vivo previously [50]. In this study, we also discovered that similar to 5′ TR of JEV, DDX3X also interacts with 5′ TR of ZIKV, albeit with lower affinity. The essential role of DDX3X in the JEV life cycle has already been established [50], but further molecular virology studies are required to investigate if DDX3X has an impact on ZIKV replication. Figure 4 suggests that both RNAs can be unwound by DDX3X, potentially indicating that DDX3X may still have a significant role to play, despite the affinity difference, in ZIKV replication. We performed an experiment (presented in Figure 4C) to simulate a knockout mutant of DDX3X, which lacks the ATP hydrolysis activity [74]. We observed that the amount of fluorescent migration appears to be reduced in the absence of ATP (Figure 4C), which can be attributed to the fact that DDX3X_132–607_ can unwind RNA but requires ATP for efficient processing of dsRNAs [78].

Previous studies for DDX5, a DDX3X homolog, have suggested that DDX5 interacts with RNA with high affinity in the nanomolar ranges. For example, fluorescence anisotropy experiments have suggested that DDX5 binds to short blunt-ended RNA duplexes with an affinity of ~230 nM [79]. A different group tested DDX5′s ability to bind short G-quadruplexes using ELISA and obtained a K_d_ of 22 nM [80]. Our work was performed using truncated DDX3X (as all our attempts to purify the full-length DDX3X in high amounts were unsuccessful), which could impact the binding specificity and affinity for RNA compared to the full-length DDX3X. Note that a truncated construct, similar to DDX3X_132–607_, was previously described as the minimally active construct [66,81]. Overall, additional work aimed at investigating the role of DDX3X in ZIKV replication could provide critical information towards potential therapeutics for these deadly viruses. In conclusion, our work provides the biochemical basis of the recognition of 5′ TRs of JEV and ZIKV by DDX3X. 

## 4. Materials and Methods

### 4.1. Overexpression and Purification of DDX3X_132–607_

The DDX3X_132–607_ cDNA construct was cloned in pET28a, followed by its transformation in Lemo21(DE3) *E. coli* cells. The cells were allowed to grow in Luria-broth containing kanamycin (50 mg/mL) and chloramphenicol (100 mg/mL) at 37 °C. The next day, the culture was transferred to Terrific broth containing 5% glycerol and grown at 37 °C for 5 h. The temperature was then lowered to 20 °C for 16–18 h. Cells were harvested via centrifugation and resuspended in lysis buffer (50 mM Tris, 500 mM NaCl, 10 mM imidazole 3 mM β-mercaptoethanol, 10 mg/mL lysozyme, 0.1% Tween-20, and 5% glycerol at pH 8.0). Following 30 min of incubation on ice, the suspension was sonicated and centrifuged at 30,000× g for 45 min. The supernatant was filtered using a 0.45 µm filter for subsequent purification using chromatography methods.

Using the ÄKTA start protein purification system (Global Life Science Solutions USA LLC, Marlborough, MA, USA) equipped with the HisTrap™ High-Performance column, we purified DDX3X_132–607_ via its hexa-histidine tag. Next, we used the ÄKTA pure purification system (Global Life Science Solutions USA LLC, Marlborough, MA, USA) with a Superdex^®^ 200 10/300 GL increase column to further purify affinity-purified protein (in 50 mM Tris, 150 mM NaCl and 3% glycerol at pH 8.0). Peak fractions representing homogenous DDX3X_132–607_ were collected and concentrated using Amicon^®^ Ultra-15 Centrifugal Filter Units (30,000 kDa molecular-wight cut-off) (Millipore Canada Ltd., Etobicoke, ON, Canada). Aliquots were stored at −80 °C. SDS-PAGE (10%) was performed by taking 10 µL of SEC-purified sample and mixed it with 2 µL SDS-loading dye and heated to 95 °C for 5 min. Following heating, samples were loaded into a 1.0 cm well PAGE casting plate (Bio-Rad Laboratories, Mississauga, ON, Canada) and ran for 1 h at 200 V in 1× SDS running buffer. A molecular weight ladder was run alongside the purified sample (unstained protein molecular weight marker 116 kDa to 14.4 kDa, Bio Basic Inc., Markham, ON, Canada). Subsequently, the gel was stained with Coomassie brilliant blue (Bio Basic Inc., Markham, ON, Canada) for visualization.

### 4.2. Preparation of Non-Coding RNAs

cDNA sequences were prepared under the control of T7 RNA polymerase, with two additional G nucleotides on the 5′ end with an XbaI restriction enzyme cut site (T^CTAGA) at the 3′ end. Then, 5′ TRs of JEV and Zika construct(s) were designed based on the Genebank sequence of KT957419.1 and KU509998.3, respectively. Underlined regions represent portions to which our fluorescent oligos were designed complementary to, described in a later section. Both RNA constructs used in the experiments are listed as follows:

1. JEV 5′ TR 1–156 (51.5 kDa, 159 nts)

5′GGAGAAGUUUUAUCGUGUGAACUUCUUGGCUUAGUAUCGUUGAGAAGAAUCGAGAGAUUAGUGCAGUUUAAACAGUUUUUUAGAACGGAAGAACAACCAUGACUAAAAAACCAGGAGGGCCCGGAAAAAACCGGGCCAUCAAUAUGCUGAAACGCGGAU3′

2. Zika 5′ TR 1–163 (52.3 kDa, 163 nts)

5′AGUUGUUACUGUUGCUGACUCAGACUGCGACAGUUCGAGUUUGAAGCGAAAGCUAGCAACAGUAUCAACAGGUUUUAUUUGGAUUUGGAAACGAGAGUUUCUGGUCAUGAAAAACCCAAAAAAGAAAUCCGGAGGAUUCCGGAUUGUCAAUAUGCUAAAACG3′

Each RNA was prepared through an in vitro transcription reaction using T7 RNA polymerase (purified in-house) followed by size-exclusion chromatography purification in 1× RNA buffer (10 mM Tris pH 7.5, 100 mM NaCl, and 5 mM MgCl_2_) using a Superdex 200 Increase GL 10/300 (Cytiva) via an ÄKTA pure FPLC (Global Life Science Solutions USA LLC, Marlborough, MA, USA). SEC peak fractions were analyzed via urea-polyacrylamide gel electrophoresis (Urea-PAGE). Then, we mixed 10 µL of each fraction with 2 µL of denaturing RNA loading dye and loaded into a 1.0 cm well PAGE casting plate (Bio-Rad Laboratories, Mississauga, ON, Canada). Urea-PAGE (7.2%) was run at room temperature, 300V, for 25 min in 0.5× TBE (Tris-Borate-EDTA) buffer, followed by staining with Sybr safe (Thermofisher Scientific, Saint-Laurant, QC, Canada) and visualization. Fractions containing a single band were deemed acceptable and used in subsequent experiments. 

### 4.3. Fluorescent Labeling of Flaviviral RNA TRs

The 5′ TRs were incubated on ice for 30 min in 0.1 M sodium acetate (pH 5.3) along with 2 mM final concentration potassium periodate. The reaction was quenched through the addition of 10 mM final concentration ethylene glycol followed by incubation on ice for 10 min. Following incubation, we performed two ethanol precipitations and resuspended the RNA in 0.1 M NaOAc and 10 mM fluorescein-5-thiosemicarbazide (FITC) and incubated the mixture in the dark and on ice for 16 h. Following fluorescent dye incubation, the mixture was phenol extracted (1 vol phenol:1 vol mixture) 5 times until the phenol layer was consistently colourless, indicating all free dye had been removed from the mixture. Finally, the resulting labelled RNA was ethanol precipitated twice and resuspended in RNA buffer. 

### 4.4. RNA–Protein Interaction Studies Using Microscale Thermophoresis

A 2-fold serial dilution was performed on DDX3X_132–607_ whereas the highest concentration was 19 µM. Next, a constant amount of fluorescent JEV or Zika 5′ TR was mixed into each serial dilution of DDX3X_132–607,_ resulting in a final concentration of 20 nM of RNA. Sample mixtures were incubated at room temperature for 10 min and then placed into Nanotemper Technologies Monolith^®^ NT.115 instrument (Nanotemper Technologies, Munich, Germany) standard capillaries and loaded into the MST. Thermophoresis was measured at room temperature (25 °C) and performed using 20% excitation power (blue filter) for both RNAs and heated using medium MST IR laser power. Fluorescent migration used to determine K_d_ was measured from 4.0 to 5.0 s and normalized to initial fluorescence (−1.0 to 0 s). Three independent replicates were merged and analyzed using MO.Affinity Analysis software v2.1.3 and fit to the standard K_d_ fit model describing a molecular interaction with a 1:1 stoichiometry according to the law of mass action. The molarity of polyU RNA could not be determined since the fragment’s lengths were variable. We used a final concentration of FITC-labeled polyU of 50 µg/mL in our negative control to achieve a similar magnitude of fluorescence. K_d_ is estimated by fitting Equation (1), where F(c) is the fraction bound at a given ligand concentration c. The unbound fraction is represented by the Fnorm signal of the target alone, and the bound fraction represents the Fnorm signal of the complex. The K_d_ is the dissociation constant and c_target_ is the final concentration of the target in the assay.

(1) $$F\left(c\right)=Unbound+\left(Bound-Unbound\right)\times \;\frac{c\;+\;c_{target}\;+\;K_{d}\;-\;\sqrt{{\left(c\;+\;c_{target}\;+\;K_{d}\right)}^{2}}\;-\;4\;c\;c_{target}}{2\;c_{target}}$$

### 4.5. Helicase Assay

We input our sequences into sfold [82] using standard conditions with no maximum distance between paired bases and no additional constraint information. The theoretical secondary structure was used to identify a portion of each RNA molecule that was highly double-stranded. DNA oligos with complementary sequences to the double-stranded region(s) of our RNA(s) were synthesized with a 5′ conjugated FITC fluorophore (Alpha DNA). The region of each RNA molecule to which the oligos hybridize is underlined and described above. The sequences for ZIKV 5′ TR and JEV 5′ TR oligo(s) are: 5′FITC/AACTGTCGCAGTCTGAGTCAGCAACAGTAACAAC and 5′FITC/TTTCCGGGCCCTCCTGGTTTTTTAGTCATGGTTGT, respectively. 

If the RNA molecule were unwound by DDX3X_132–607_, it would create an opportunity for the oligos to hybridize to the 5′ TR RNA. The reaction mixture contains 20 nM of FITC-DNA oligos, 1 µM of the RNA, and 4.25 mM of ATP. DDX3X_132–607_ is added to a final concentration of 10 µM. We used BSA at the same concentration as a control for DDX3X. The ATP dependence assay involved comparing 10 µM DDX3X_132–607_ with 20 nM of FITC-DNA oligos and 1 µM of the RNA, but one set of capillaries contained 4.25 mM of ATP and another set did not. An experiment consists of 3 sets of 4 capillaries for each sample, which can then be compared to detect a change in fluorescent migration because of a binding event. Our data represent the normalized magnitude of fluorescent migration differences between two sets of assay conditions. Data were processed using MO.Affinity software, which assesses the signal to noise ratio between a run with and without the protein. Signal to noise is a measure of the response amplitude that is divided by the noise of the environment, as presented in Equation (2) [83]. If the signal to noise ratio rises above 5, the assay indicates that a binding event has occurred, and the ration >12 suggests that the assay is considered as desirable [83,84].

(2) $$S/N=\;\frac{Response\;Amplitude}{\sqrt{\frac{\sum_{i}{\left(r_{i}\;-\;\bar{r}\right)}^{2}}{n\;-\;1}}}$$

## Data Availability

All data has been presented in this article.

## Supplementary Materials

The Supplementary Materials are available online at https://www.mdpi.com/1422-0067/22/1/413/s1.

## Abbreviations

MST	microscale thermophoresis
SEC	size-exclusion chromatography
PAGE	polyacrylamide gel electrophoresis
JEV	Japanese encephalitis virus
ZIKV	Zika virus
TR	terminal regions
