# Supplementary material for: Human DDX3X Unwinds Japanese Encephalitis and Zika Viral 5′ Terminal Regions
Source: Int J Mol Sci. 2021 Jan 2;22(1):413. doi: 10.3390/ijms22010413 (PMC7795613; doi:10.3390/ijms22010413)
Supplement: Supplementary file 1 [file ijms-22-00413-s001.pdf]

Supplementary Materials

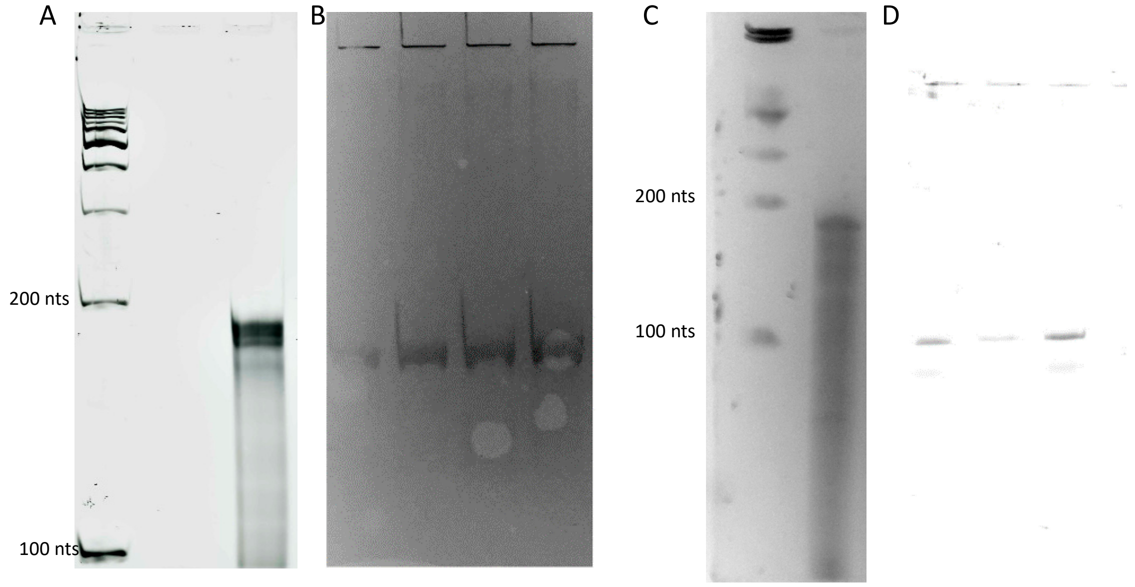

Supplementary Figure S1. Urea PAGEs representing Flaviviral RNAs. (**A**) *In vitro* transcribed and (**B**) SEC-purified ZIKA 5′ TR, fractions containing a single band were used for downstream experiments (7.2% Urea PAGE ran for 30 min, 300 V in 0.5x TBE running buffer). (**B)** 5′ TR ZIKV RNA after IVT and SEC purification. 7.5% Urea-PAGE at 300 V for 30 min in 1x TBE running buffer (**C**) *In vitro* transcribed and (**D**) SEC-purified JEV 5′ TR suggesting that the RNA preparation is pure (7.5% Urea-PAGE, 300 V, 20 min, 1x TBE running buffer). Note, that the JEV (159 nts) and ZIKV (163 nts) RNA are right below the 200 nts DNA ladder in the Urea-PAGEs. The DNA ladder does not provide an analytical measurement of exactly where the RNA should be relative to the ladder but gives a comparable location to ensure that the RNA is pure and is close to its respective length.
